# Correlation of Leukocyte Telomere Length Measurement Methods in Patients with Dyskeratosis Congenita and in Their Unaffected Relatives

**DOI:** 10.3390/ijms18081765

**Published:** 2017-08-13

**Authors:** Payal P. Khincha, Casey L. Dagnall, Belynda Hicks, Kristine Jones, Abraham Aviv, Masayuki Kimura, Hormuzd Katki, Geraldine Aubert, Neelam Giri, Blanche P. Alter, Sharon A. Savage, Shahinaz M. Gadalla

**Affiliations:** 1 Clinical Genetics Branch, Division of Cancer Epidemiology and Genetics, National Cancer Institute, National Institutes of Health, Bethesda, MD 20892, USA; payal.khincha@nih.gov (P.P.K.); girin@mail.nih.gov (N.G.); alterb@mail.nih.gov (B.P.A.); savagesh@mail.nih.gov (S.A.S.); 2 Cancer Genomics Research Laboratory, Division of Cancer Epidemiology and Genetics, National Cancer Institute, National Institutes of Health, Bethesda, MD 20892, USA; dagnallc@mail.nih.gov (C.L.D.); hicksbel@mail.nih.gov (B.H.); kristine.jones@nih.gov (K.J.); 3 Cancer Genomics Research Laboratory, Leidos Biomedical Research, Inc., Frederick National Laboratory for Cancer Research, Frederick, MD 21701, USA; 4 Center of Human Development and Aging, Rutgers State University of New Jersey, Newark, NJ 07103, USA; avivab@njms.rutgers.edu (A.A.); kimurama@njms.rutgers.edu (M.K.); 5 Biostatistics Branch, Division of Cancer Epidemiology and Genetics, National Cancer Institute, National Institutes of Health, Bethesda, MD 20892, USA; katkih@mail.nih.gov; 6 Terry Fox Laboratory, British Columbia Cancer Agency, Vancouver, BC V5Z 1L3, Canada; gaubert@bccrc.ca

**Keywords:** telomere length measurement, qPCR, Southern blot, flow FISH, correlation, dyskeratosis congenita

## Abstract

Several methods have been employed to measure telomere length (TL) in human studies. It has been difficult to directly compare the results from these studies because of differences in the laboratory techniques and output parameters. We compared TL measurements (TLMs) by the three most commonly used methods, quantitative polymerase chain reaction (qPCR), flow cytometry with fluorescence in situ hybridization (flow FISH) and Southern blot, in a cohort of patients with the telomere biology disorder dyskeratosis congenita (DC) and in their unaffected relatives (controls). We observed a strong correlation between the Southern blot average TL and the flow FISH total lymphocyte TL in both the DC patients and their unaffected relatives (*R*^2^ of 0.68 and 0.73, respectively). The correlation between the qPCR average TL and that of the Southern blot method was modest (*R*^2^ of 0.54 in DC patients and of 0.43 in unaffected relatives). Similar results were noted when comparing the qPCR average TL and the flow FISH total lymphocyte TL (*R*^2^ of 0.49 in DC patients and of 0.42 in unaffected relatives). In conclusion, the strengths of the correlations between the three widely used TL assays (qPCR, flow FISH, and Southern blot) were significantly different. Careful consideration is warranted when selecting the method of TL measurement for research and for clinical studies.

## 1. Introduction

Telomeres are nucleoprotein complexes consisting of tandem nucleotide repeats (TTAGGG)_n_ and a protein complex at the ends of eukaryotic chromosomes. They are essential for chromosomal stability, and shorten with each cell division [1,2]. Telomere length (TL) is heritable and is highly variable, depending on the individual’s age, tissue type, and possibly lifestyle factors [3,4,5,6,7]. It has been extensively studied in association with age-related diseases, such as cardiovascular disease and cancer, as well as with lifespan and mortality (reviewed in [8]).

Dyskeratosis congenita (DC) is an inherited bone marrow failure (BMF) and cancer predisposition syndrome caused by germline mutations in telomere biology genes [1,9]. Patients with DC have a very short TL for their age, defined by a TL of less than the first percentile for their age, as measured by flow cytometry and fluorescence in situ hybridization (flow FISH) [10,11].

Several TL measurement (TLM) methods, including Southern blot, flow FISH, and quantitative polymerase chain reaction (qPCR), have been developed for use in a variety of clinical and research settings. The selection of the TLM method is usually contingent on the research question and study population. Each TLM method has its own strengths and limitations that have been reviewed in detail elsewhere [12,13]. Briefly, terminal restriction fragment (TRF) measurement by Southern blot uses restriction enzymes to cut DNA at the subtelomere. It measures the size of resolved telomere DNA fragments and the subtelomeric region up to the nearest restriction site, in kilobases (kb) [14]. TLMs by Southern blot are labor intensive, require relatively large amounts of DNA (2–3 μg/run) and demand considerable expertise to obtain accurate and reliable results [14]. The qPCR-based method determines the relative telomeric DNA content in a given sample, expressed as a ratio of the amplified telomeric repeats to amplified repeats of a single copy gene (T/S: telomeric/single copy gene (36B4) PCR products). This technique is widely used in large epidemiologic studies because of its high-throughput nature and small DNA requirements (~20 ng/sample). However, qPCR relative TL (RTL) measurements have not been optimized across laboratories, and they are limited by its relatively high measurement error and low sensitivity to identifying individuals with a very short TL, including those patients with DC [15,16]. Flow FISH allows for the measurement of TL in leukocyte subsets but requires viable cells. It is labor intensive, is performed by few laboratories across the world, and is relatively expensive [12,17]. A flow FISH TL study of more than 800 healthy individuals showed differences in TL by leukocyte subsets [18], which may affect the average measures of TL in DNA from leukocytes by Southern blot or qPCR. This disparity is especially important in TL studies of diseases for which the leukocyte differential counts at the time of the TLM may be different from those of healthy individuals, such as in immunodeficiency or BMF disorders. In this study, we compared the average TL measured by qPCR or Southern blot and the total lymphocyte TL measured by flow FISH in a cohort of patients with DC and in their unaffected relatives.

## 2. Results

The study included 35 patients with DC and 53 unaffected relatives from 36 families (14 families contributed only DC cases, 13 contributed only unaffected relatives and 9 contributed both). The participant characteristics are summarized in Table 1. Briefly, patients with DC were younger than their relatives (median age of 27 years and range of 5–70 years vs median age of 41 years and range of 3–69 years, respectively; *p* < 0.05), and more likely to be males (63% vs 43% male in patients and relatives, respectively; *p* = 0.7). Twenty-seven patients (77%) had a pathogenic germline variant in a known causative DC gene (5 *DKC1*, 5 *TINF2*, 6 *TERT*, 9 *TERC*, 1 *WRAP53* and 1 *RTEL1*).

All three TLM methods showed an inverse association between TL and age in the DC patients (*r* of −0.29, −0.23 and −0.31 for qPCR, Southern blot and flow FISH, respectively) and in unaffected relatives (*r* of −0.12, −0.50 and −0.37 for qPCR, Southern blot and flow FISH, respectively). As expected, the patients with DC had significantly shorter TL than their unaffected relatives using any of the three methods (*p* < 0.001; Table 1). The mean inter-assay coefficient of variation (CV: standard deviation/mean) of Southern blot was 1.8%, and the intra-class correlation (ICC: variance between duplicates/total variance across samples) was 0.99, as compared with a CV of 5.13% and an ICC of 0.92 for the qPCR assay.

### 2.1. Telomere Length Measurement (TLM) Correlations in Patients with Dyskeratosis Congenita (DC)

**Figure 1:**
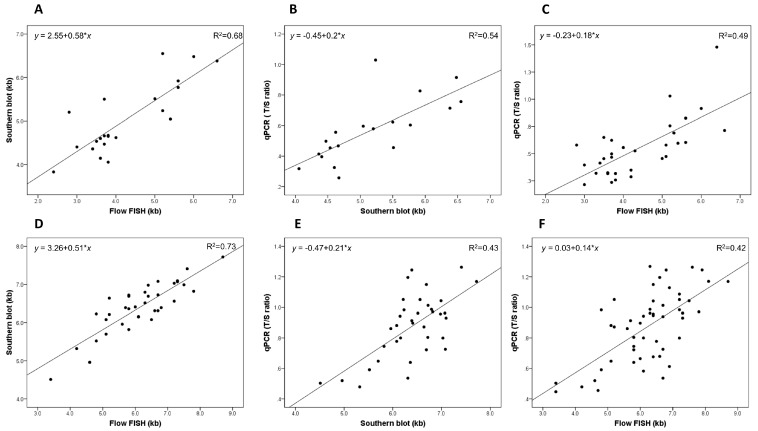
Correlation of telomere length measurements (TLMs) between quantitative polymerase chain reaction (qPCR), total lymphocytes by flow cytometry with fluorescence in situ hybridization (flow FISH) and Southern blot in patients with dyskeratosis congenita (DC) and in their unaffected relatives; kb: kilobases. (**A**) Correlation between flow FISH and Southern blot in patients with DC; (**B**) Correlation between qPCR and Southern blot in patients with DC; (**C**) Correlation between qPCR and flow FISH in patients with DC; (**D**) Correlation between flow FISH and Southern blot in unaffected relatives; (**E**) Correlation between qPCR and Southern blot in unaffected relatives and (**F**) Correlation between qPCR and flow FISH in unaffected relatives.

We noted statistically significant correlations between all three methods in the patients with DC, and the strongest was noted between the Southern blot average TL and the flow FISH total lymphocyte TL (*R*^2^ = 0.68; 95% CI = 0.46–0.87; *p* < 0.001). The *R*^2^ value for the association between the qPCR RTL and the Southern blot average TL was 0.54 (95% CI = 0.27–0.81; *p* < 0.001), and that between the qPCR RTL and the flow FISH total lymphocyte TL was 0.49 (95% CI = 0.26–0.72; *p* < 0.001); see Figure 1A–C.

### 2.2. TLM Correlations in Unaffected Relatives

The correlation between the average TL by Southern blotting and the total lymphocyte TL by flow FISH was strong (*R*^2^ = 0.73; 95% CI = 0.60–0.88; *p* < 0.001; Figure 1D). Modest correlations were noted between the qPCR RTL and the Southern blot average TL (*R*^2^ = 0.43; 95% CI = 0.2–0.66; *p* < 0.001) or the total lymphocyte TL by flow FISH (*R*^2^ = 0.42; 95% CI = 0.22–0.62; *p* < 0.001; Figure 1E,F).

The correlations between the qPCR RTL or Southern blot average TL and the lymphocyte cell type-specific TL by flow FISH in the DC patients and their relatives are presented in Table S1.

## 3. Discussion

Our analyses showed that the correlations between TL measured by Southern blot and flow FISH were stronger than those between qPCR and either Southern blot or flow FISH. This was true in both the patients with DC and in their unaffected relatives.

Studies comparing the TLMs are challenging because of differences in laboratory techniques and pre-measurement factors [12]. The qPCR RTL values are affected by the DNA extraction method, by the well position, and possibly by other pre-assay technical factors that are not yet well-defined [19,20,21,22]. Here, we showed modest correlations between the qPCR RTL and the Southern blot average TL (*R*^2^ of 0.54 in patients with DC and of 0.43 in their unaffected relatives) or the flow FISH TL (*R*^2^ of 0.49 in patients with DC and of 0.42 in their unaffected relatives). Previous studies comparing the TLM by qPCR and Southern blot report a wide range of *R*^2^ (between 0.27 and 0.83) [16,23]. The differences may be explained by the sample size, technical aspects such as the DNA quality, the sample preparation, and/or variation in the range of TL in the sample (a larger range yields a higher *R*^2^). Notably, a higher inter-laboratory variability in TLMs has been reported with qPCR than Southern blot in previous studies [20,24].

In agreement with our results, a previous study comparing the same three assays in patients with BMF or idiopathic pulmonary fibrosis and in healthy controls showed the highest measurement correlations between the flow FISH leukocyte TL and the TRF by Southern blot (*R*^2^ of 0.60 in controls and of 0.51 in patients) [25]. The same study reported a modest correlation between the qPCR RTL and the leukocyte flow FISH TL (*R*^2^ = 0.33) in healthy controls, but no correlation was noted in patients (*R*^2^ = 0.1). The stronger correlation between flow FISH and Southern blot compared with flow FISH and qPCR in our study may have been explained by a higher measurement precision for the Southern blot assay (CV of 1.8% for Southern blotting and of 5.1% for qPCR). A previous study comparing the Southern blot average TL and qPCR RTL measurements showed similar results (CV of 1.7% and 6.4%, respectively) [16]. While laboratory quality control measures such as the CV and ICC validate the reproducibility of qPCR, readers are cautioned not to interpret these results as a surrogate for accuracy in the diagnostic abilities of each technique in the context of DC and related telomere biology disorders, as we have previously reported [15].

Our study showed a negative correlation between TL and age for all three methods. However, the strength of this correlation varied with the assay type (*R*^2^ = 0.02 for qPCR, *R*^2^ = 0.14 for total lymphocyte flow FISH, and *R*^2^ = 0.26 for Southern blotting when analyzed in unaffected relatives). A previous study showed that age accounted for 17% and 29% of the variation in the leukocyte TL by the qPCR and Southern blot methods, respectively [16]. It is important to note that while the correlations with age may be different for each method, the relationship between TL and age is traditionally calculated from a linear regression, while curvilinear models have also been tested in other studies [16,22,26].

In conclusion, our study showed variable correlations between TL measured by qPCR, flow FISH, and Southern blot, and the strongest associations were noted between the flow FISH and Southern blot TL. These correlation data and those of other published studies may not be applicable to assays performed in different laboratories. The wide range of reported method correlations in different studies highlights the importance of performing laboratory-specific internal validations. Our data show that flow FISH and Southern blot TL assays provide more reliable measurements. Studies aiming to understand the sources of variability and to improve the qPCR TL assay are warranted as this assay is the best suited for large epidemiological studies.

## 4. Materials and Methods

### 4.1. Study Participants

Patients with DC and their unaffected family members were enrolled in the National Cancer Institute’s (NCI’s) Institutional Review Board-approved inherited BMF syndromes (IBMFS) study (NCI 02-C-0052, NCT00027274; www.marrowfailure.cancer.gov) [27]. Patients were classified as having DC if they had a pathogenic germline variant in one of the known DC genes, or if they had at least two features of the diagnostic triad and other clinical findings consistent with DC, such as physical findings or hematologic or neoplastic complications [28]. Unaffected relatives included in this study were family members of the DC patients who had no clinical features suggestive of DC and who had tested negative for the known pathogenic variant causative of DC in their family. Family members of the DC patients with a yet unknown genetic cause of disease were excluded from this study.

### 4.2. DNA Extraction

DNA for the qPCR and Southern blot assays was extracted from whole blood samples. The methods used included the Organic, Puregene and Qiagen Autopure methods.

### 4.3. Quantitative Polymerase Chain Reaction (qPCR) Assay

We used a monoplex qPCR assay adapted from previously described methods [26]; details are available elsewhere [29]. Briefly, TL was measured in triplicate and expressed as a ratio of the amplified telomeric repeats to amplified repeats of a single copy gene (T/S: telomeric/single copy gene (36B4) PCR products). The final measurements were exponentiated and were corrected for a reference sample. The mean inter-assay CV was 0.61% for the telomere assay and 0.60% for the 36B4 assay. The inter-assay CV for the standardized T/S measure from duplicates was 5.13%, and the ICC was 92%.

### 4.4. Southern Blot Assay

The average TLM was obtained from the TRF analysis by Southern blot, as described previously [14,29]. Briefly, DNA was digested with the restriction enzymes *HinfI* (10 U) and *RsaI* (10 U; Roche). The digested DNA samples and DNA ladders were resolved on 0.5% agarose gels. The DNA was then depurinated, denatured and neutralized. The DNA was transferred for 1 h to a positively charged nylon membrane (Roche), and was hybridized at 65 °C with the Digoxigenin (DIG)-labeled telomeric probe overnight in 5x Saline Sodium Citrate (SSC), 0.1% Sarkosyl, 0.02% Sodium Dodecyl Sulphate (SDS) and 1% blocking reagent (Roche, Indianapolis, IN, USA). The DNA was washed three times at room temperature in 2x SSC and 0.1% SDS, each for 15 min, and once in 2x SSC for 15 min. The DIG-labeled probe was detected by the DIG luminescent detection kit (Roche) and was exposed on an X-ray film. All autoradiographs were scanned, and the TRF signal was digitized. The optical density values versus DNA migration distances were converted to the optical density (adjusted for background)/molecular weight versus the molecular weight. Each set of samples was run on duplicates resolved on different gels. The mean TRF length was computed with the following equation:

Mean TRF length = Σ(OD*i*)/Σ(OD*i*/L*i*), where OD*i* is the optical density at position *i* and L*i* is the TRF length at position *i*.

The inter-assay CV of the TRF samples was 1.8%, and the ICC was 99%.

### 4.5. Flow cytometry with fluorescence in situ hybridization (Flow FISH)

This method uses isolated leukocytes from fresh whole blood; a detailed description of the method is available elsewhere [17]. Briefly, leukocytes were isolated by osmotic lysis of erythrocytes from whole blood using ammonium chloride. The leukocytes were then mixed with bovine thymocytes of known TL (internal control), denatured in formamide at 87 °C, hybridized with a fluorescein-conjugated (CCCTAA)_3_ peptide nucleic acid (PNA) probe specific for telomere repeats and counterstained with LDS751 DNA dye. The leukocytes were sorted into granulocytes and lymphocytes on the basis of light scattering and the fluorescence intensity. The lymphocytes were further characterized into subsets defined by labeled antibodies specific for CD20, CD45RA and CD57 relative to internal control cells, and the unstained controls were measured on a FACS Calibur instrument. The values of the total lymphocyte TL were used for the main analysis.

### 4.6. Statistical Analysis

We used generalized estimating equations to compare TL in DC patients and their relatives to account for TL correlations within families; the models were adjusted for age and sex. The correlation between TL and age was calculated using Pearson’s correlation coefficient. We used linear regression models to evaluate the strength of the association between the TLMs generated by the three methods; the models were stratified by the participant disease status (DC patients and unaffected relatives). All the analyses were two sided, and a *p*-value of <0.05 was considered statistically significant. We used Excel software (Microsoft, 2007 release) and SPSS Statistics version 21 (IBM Corp. Armonk, NY, USA) for all the analyses.

## 5. Conclusions

Our study showed variable correlations between TL measured by qPCR, flow FISH, and Southern blot, and the strongest associations were noted between the flow FISH and Southern blot TL. These correlation data and those of other published studies may not be applicable to assays performed in different laboratories. The wide range of reported method correlations in different studies highlights the importance of performing laboratory-specific internal validations. Our data show that flow FISH and Southern blot TL assays provide more reliable measurements. Studies aiming to understand the sources of variability and to improve the qPCR TL assay are warranted as this assay is the best suited for large epidemiological studies.

## Figures and Tables

**Table 1:** Demography and comparison of telomere length (TL) by different methods.

Header	DC (*n* = 35)	Unaffected Relatives (*n* = 53)
Age in years, median (range)	27 (5–70)	41 (3–69)
Gender, male:female	2:1	1:1
Genetic Mutation Number (%)		
*TERC*	9 (26%)	Pathogenic variant(s) causative of DC in the family were not present
*TERT*	6 (17%)	
*DKC1*	5 (14%)	
*TINF2*	5 (14%)	
*WRAP53*	1 (3%)	
*RTEL1*	1 (3%)	
Unknown gene	8 (23%)	
TL Median (Range)		
qPCR (T/S ratio) *	0.50 (0.21–1.48)	0.91 (0.45–1.27)
Flow FISH lymphocytes (kb) *	3.8 (2.4–6.6)	6.4 (3.4–8.7)
Southern blot (kb) *	4.6 (3.8–6.6)	6.4 (4.5–7.7)

* *p*-value < 0.001 from generalized estimating equations comparing TL in dyskeratosis congenita (DC) and unaffected relatives and adjusted for age and sex.

## Supplementary Materials

Supplementary materials can be found at www.mdpi.com/1422-0067/18/8/1765/s1.

## References

[1] Ballew B.J., Savage S.A. (2013). Updates on the biology and management of dyskeratosis congenita and related telomere biology disorders. Expert. Rev. Hematol..

[2] De Lange T., Shiue L., Myers R.M., Cox D.R., Naylor S.L., Killery A.M., Varmus H.E. (1990). Structure and variability of human chromosome ends. Mol. Cell. Biol..

[3] Sanders J.L., Newman A.B. (2013). Telomere length in epidemiology: A biomarker of aging, age-related disease, both, or neither?. Epidemiol. Rev..

[4] Aubert G., Lansdorp P.M. (2008). Telomeres and aging. Physiol. Rev..

[5] Gadalla S.M., Cawthon R., Giri N., Alter B.P., Savage S.A. (2010). Telomere length in blood, buccal cells, and fibroblasts from patients with inherited bone marrow failure syndromes. Aging.

[6] Aston K.I., Hunt S.C., Susser E., Kimura M., Factor-Litvak P., Carrell D., Aviv A. (2012). Divergence of sperm and leukocyte age-dependent telomere dynamics: Implications for male-driven evolution of telomere length in humans. Mol. Hum. Reprod..

[7] Cassidy A., De V.I., Liu Y., Han J., Prescott J., Hunter D.J., Rimm E.B. (2010). Associations between diet, lifestyle factors, and telomere length in women. Am. J. Clin. Nutr..

[8] Stone R.C., Horvath K., Kark J.D., Susser E., Tishkoff S.A., Aviv A. (2016). Telomere length and the cancer-atherosclerosis trade-off. PLoS Genet..

[9] Dokal I. (2011). Dyskeratosis congenita. Hematology Am. Soc. Hematol. Educ. Program.

[10] Alter B.P., Baerlocher G.M., Savage S.A., Chanock S.J., Weksler B.B., Willner J.P., Peters J.A., Giri N., Lansdorp P.M. (2007). Very short telomere length by flow fluorescence in situ hybridization identifies patients with dyskeratosis congenita. Blood.

[11] Alter B.P., Rosenberg P.S., Giri N., Baerlocher G.M., Lansdorp P.M., Savage S.A. (2012). Telomere length is associated with disease severity and declines with age in dyskeratosis congenita. Haematologica.

[12] Aubert G., Hills M., Lansdorp P.M. (2012). Telomere length measurement-caveats and a critical assessment of the available technologies and tools. Mutat. Res..

[13] Baerlocher G.M., Mak J., Tien T., Lansdorp P.M. (2002). Telomere length measurement by fluorescence in situ hybridization and flow cytometry: Tips and pitfalls. Cytometry.

[14] Kimura M., Stone R.C., Hunt S.C., Skurnick J., Lu X., Cao X., Harley C.B., Aviv A. (2010). Measurement of telomere length by the southern blot analysis of terminal restriction fragment lengths. Nat. Protoc..

[15] Gadalla S.M., Khincha P.P., Katki H.A., Giri N., Wong J.Y., Spellman S., Yanovski J.A., Han J.C., de Vivo I., Alter B.P. (2016). The limitations of qPCR telomere length measurement in diagnosing dyskeratosis congenita. Mol. Genet. Genomic Med..

[16] Aviv A., Hunt S.C., Lin J., Cao X., Kimura M., Blackburn E. (2011). Impartial comparative analysis of measurement of leukocyte telomere length/DNA content by southern blots and qPCR. Nucleic Acids Res..

[17] Baerlocher G.M., Vulto I., De J.G., Lansdorp P.M. (2006). Flow cytometry and fish to measure the average length of telomeres (flow FISH). Nat. Protoc..

[18] Aubert G., Baerlocher G.M., Vulto I., Poon S.S., Lansdorp P.M. (2012). Collapse of telomere homeostasis in hematopoietic cells caused by heterozygous mutations in telomerase genes. PLoS Genet..

[19] Cunningham J.M., Johnson R.A., Litzelman K., Skinner H.G., Seo S., Engelman C.D., Vanderboom R.J., Kimmel G.W., Gangnon R.E., Riegert-Johnson D.L. (2013). Telomere length varies by DNA extraction method: Implications for epidemiologic research. Cancer Epidemiol. Biomarkers Prev..

[20] Verhulst S., Susser E., Factor-Litvak P.R., Simons M.J., Benetos A., Steenstrup T., Kark J.D., Aviv A. (2015). Commentary: The reliability of telomere length measurements. Int. J. Epidemiol..

[21] Raschenberger J., Lamina C., Haun M., Kollerits B., Coassin S., Boes E., Kedenko L., Kottgen A., Kronenberg F. (2016). Influence of DNA extraction methods on relative telomere length measurements and its impact on epidemiological studies. Sci. Rep..

[22] Eisenberg D.T., Kuzawa C.W., Hayes M.G. (2015). Improving qPCR telomere length assays: Controlling for well position effects increases statistical power. Am. J. Hum. Biol..

[23] Elbers C.C., Garcia M.E., Kimura M., Cummings S.R., Nalls M.A., Newman A.B., Park V., Sanders J.L., Tranah G.J., Tishkoff S.A. (2013). Comparison between southern blots and qPCR analysis of leukocyte telomere length in the health abc study. J. Gerontol. A Biol. Sci. Med. Sci..

[24] Martin-Ruiz C.M., Baird D., Roger L., Boukamp P., Krunic D., Cawthon R., Dokter M.M., van der Harst P., Bekaert S., de Meyer T. (2015). Reproducibility of telomere length assessment: Authors’ response to damjan krstajic and ljubomir buturovic. Int. J. Epidemiol..

[25] Gutierrez-Rodrigues F., Santana-Lemos B.A., Scheucher P.S., Alves-Paiva R.M., Calado R.T. (2014). Direct comparison of flow-fish and qPCR as diagnostic tests for telomere length measurement in humans. PLoS ONE.

[26] Cawthon R.M. (2002). Telomere measurement by quantitative pcr. Nucleic Acids Res..

[27] Alter B.P., Giri N., Savage S.A., Peters J.A., Loud J.T., Leathwood L., Carr A.G., Greene M.H., Rosenberg P.S. (2010). Malignancies and survival patterns in the national cancer institute inherited bone marrow failure syndromes cohort study. Br. J. Haematol..

[28] Vulliamy T.J., Marrone A., Knight S.W., Walne A., Mason P.J., Dokal I. (2006). Mutations in dyskeratosis congenita: Their impact on telomere length and the diversity of clinical presentation. Blood.

[29] Gadalla S.M., Wang T., Dagnall C., Haagenson M., Spellman S.R., Hicks B., Jones K., Katki H.A., Lee S.J., Savage S.A. (2016). Effect of recipient age and stem cell source on the association between donor telomere length and survival after allogeneic unrelated hematopoietic cell transplantation for severe aplastic anemia. Biol. Blood Marrow Transplant..

