# Supplementary material for: Correlation of Leukocyte Telomere Length Measurement Methods in Patients with Dyskeratosis Congenita and in Their Unaffected Relatives
Source: Int J Mol Sci. 2017 Aug 13;18(8):1765. doi: 10.3390/ijms18081765 (PMC5578154; doi:10.3390/ijms18081765)
Supplement: Supplementary file 1 [file ijms-18-01765-s001.pdf]

Supplementary Table S1. Correlations between qPCR relative telomere length (T/S ratio), Southern blot average telomere length (kilobases) and lymphocyte cell-specific telomere length by flow FISH (kilobases).

Telomere length measurement method	Disease Status	Cell-Specific Lymphocyte TL by flow FISH			
		CD45+ lymphocytes	CD45- lymphocytes	CD20+ lymphocytes	CD57+ lymphocytes
		R^2			
qPCR	DC	0.41	0.50	0.48	0.53
Southern Blot		0.63	0.55	0.81	0.53
qPCR	Relatives	0.42	0.33	0.53	0.20
Southern Blot		0.79	0.61	0.73	0.55
